# Impacts of Citric Acid and Malic Acid on Fermentation Quality and Bacterial Community of Cassava Foliage Silage

**DOI:** 10.3389/fmicb.2020.595622

**Published:** 2020-12-14

**Authors:** Mao Li, Lidong Zhang, Qing Zhang, Xuejuan Zi, Renlong Lv, Jun Tang, Hanlin Zhou

**Affiliations:** ^1^Tropical Crops Genetic Resources Institute, Chinese Academy of Tropical Agricultural Sciences, Danzhou, China; ^2^Key Laboratory of Ministry of Education for Genetics and Germplasm Innovation of Tropical Special Trees and Ornamental Plants, Key Laboratory of Germplasm Resources of Tropical Special Ornamental Plants of Hainan Province, College of Forestry, College of Tropical Crops, Hainan University, Danzhou, China; ^3^Guangdong Province Research Center of Woody Forage Engineering Technology, Guangdong Research and Development Centre of Modern Agriculture (Woody Forage) Industrial Technology, Guangdong Key Laboratory for Innovative Development and Utilization of Forest Plant Germplasm, State Key Laboratory for Conservation and Utilization of Subtropical Agro-Bioresources, Integrative Microbiology Research Centre, College of Forestry and Landscape Architecture, South China Agricultural University, Guangzhou, China

**Keywords:** cassava foliage, citric acid, malic acid, lactic acid bacteria, silage fermentation, bacterial community

## Abstract

The microbiota and fermentation quality of cassava foliage (CF) ensiled in the absence of additive (CK), or the presence of citric acid (CA), malic acid (MA), and their combination with a *Lactobacillus plantarum* strain (CAL and MAL)were investigated. These additives reduced (*P* < 0.05) the pH, butyric acid, and ammonia-N contents but increased (*P* < 0.05) the lactic acid content, and CAL and MAL showed similar remarkable effects. *Paenibacillus* (mean, 27.81%) and *Bacillus* (mean, 16.04%) were the predominant strains in CF silage. The addition of CA or MAL increased the abundance of *Paenibacillus* (25.81–52.28% and 47.97%, respectively), and the addition of MA increased the abundance of *Bacillus* (15.76–32.48%) compared with the CK group. Moreover, CAL and MAL increased the abundances of the potentially desirable bacteria *Cellulosimicrobium* (CAL 0–12.73%), *Hyphomicrobium* (0–7.90% and 8.94%), and *Oceanobacillus* (0–8.37% and 3.08%) compared with the CK group. These findings suggested that CA and MA could enhance the silage quality of CF, and their combinations with *Lactobacillus plantarum* were more effective.

## Introduction

As a major food or bio-energy crop, cassava (*Manihot esculenta* Crantz) is widely cultivated in tropical areas, cassava industry in China has been rapidly developed in recent years, and the total production of cassava foliage (CF) in China is 3,000,000 tons per year (Li et al., 2019a,c). Burning or discarding is the traditional way to dispose of CF, leading to serious environmental pollution. The use of cassava by-products for animal feed is one of the alternative approaches to overcome this problem (Napasirth et al., 2015; Li et al., 2019b). CF contains abundant nutrients, especially high levels of crude protein (CP) (16.41–22.68%). CF is widely used in animal feed in many countries, and it has great potential for improving the production performance of animals (Régnier et al., 2013; Napasirth et al., 2015; Li et al., 2017a, 2020). Besides, CF harvest is seasonal, with higher quantity biomass in summer or rainy season. However, its supply decreases in the winter, which needs proper pretreatment for long-term preservation.

Ensiling is considered the best processing approach to maintaining green forage by lactic acid bacteria (LAB) fermentation under anaerobic conditions. It is difficult to make high-quality silage using CF alone due to its lower content of water-soluble carbohydrate (WSC) (Napasirth et al., 2015). Therefore, the fermentation quality of CF silage remains poor in the absence of additives. LAB is one of the crucial factors for high-quality silage, and LAB inoculants are widely adopted for silage processing (Cai et al., 1998; Napasirth et al., 2015; Li et al., 2017b, 2019a; Wang et al., 2019). Citric acid (CA) and malic acid (MA) are two types of antioxidants that are also safe additives, which are widely used in food, medicine, daily chemical, and health product industries (Ke et al., 2017). Besides, CA and MA can be used as a carbohydrate source to provide energy for microbial activities, which accelerates the LAB growth. Furthermore, CA and MA can also effectively reduce the pH and inhibit the growth of yeast and mold during ensiling. Therefore, in recent years, CA and MA have been considered as ideal and novel silage additives for improving the fermentation quality (Li et al., 2016; Ke et al., 2017; Ke W. C. et al., 2018; Ke W. et al., 2018; He et al., 2020; Lv et al., 2020). Besides, Ke W. et al. (2018) found that CA or MA, in combination with LAB inoculants, has positive effects on alfalfa silage. However, the impacts of CA, MA, and LAB inoculants on the microbial community of CF silage remain largely unexplored.

In the present study, we hypothesized that CA, MA, and LAB had beneficial effects on the fermentation and microbial community of CF silage, and there might be a potential synergistic effect when CA and MA are combined with LAB. Therefore, we attempted to explore the impacts of CA, MA, and LAB inoculants on bacterial community and fermentation characteristics of CF silage.

## Materials and Methods

### Silage Preparation

Cassava was provided by the experimental base of the Chinese Academy of Tropical Agricultural Sciences (109°58′E, 19°52′N). The CF was harvested and sectioned into small pieces (about 2 cm). Six different treatments were conducted in the present work: control (no additives, CK), LAB inoculants (*Lactobacillus plantarum*, LAB), MA, MA in combination with *Lactobacillus plantarum* (MAL), CA, and CA in combination with *Lactobacillus plantarum* (CAL). Each treatment was carried out in triplicate. The application rate of LAB was 1.0 × 10^5^ colony-forming units (CFU)/g of fresh matter (FM), and that of MA and CA was 5 g/kg of FM. Briefly, 200 g of CF was mixed with additives, and the mixture was placed into plastic bags (30 cm × 10 cm × 4 cm; Menghua Packing Co., Ltd., Guangzhou, China), followed by incubation at room temperature (25–30°C). The ensiling process lasted for 30 days, and then chemical composition, organic acid, and microbial community were examined.

### Chemical and Microbial Compositional Analysis

Specimens were dried at 65°C for 2 days and passed through a 1.0 mm sieve before the chemical assay. The contents of dry matter (DM), CP, organic matter (OM), and ether extracts (EE) were examined according to previously established approaches (AOAC, 1990). Moreover, the contents of neutral detergent fiber (NDF) and acid detergent fiber (ADF) were assessed using a previously established method (Van Soest et al., 1991). Heat-stable amylase and sodium sulfite were adopted in the determination of NDF. WSC was determined according to a previously described method (Murphy, 1958). The fermented silages were assayed using cold-water extracts. Briefly, 50 g wet silage was blended with 200 mL distilled water, followed by overnight incubation at 4°C and filtration. The pH and contents of organic acids (lactic acid, acetic acid, propionic acid, and butyric acid) and ammonia-N were assessed using previously established approaches (Li et al., 2019d).

The microbial composition was analyzed according to a previously described method (Wang et al., 2019). Briefly, 20 g silage samples were blended with 180 mL sterilized saline, and then LAB, coliform, yeasts, and molds were enumerated on Man Rogosa Sharpe (MRS) agar, Violet Red Bile agar, and Rose Bengal agar, respectively.

### Microbial Diversity Analysis

#### DNA Extraction and 16S rRNA Gene Sequencing

The above-mentioned extracts were used for the molecular analysis of the microbiota. Microbial DNA was isolated from silage specimens using the E.Z.N.A.^®^ soil DNA Kit (Omega Bio-Tek, Norcross, GA, United States) according to the manufacturer’s instructions. The concentration and purity of extracted DNA were assessed by a NanoDrop 2000 UV-vis spectrophotometer (Thermo Scientific, Wilmington, DE, United States), and DNA integrity was confirmed by electrophoresis on 1% agarose gel. The V3-V4 hypervariable region of the bacterial 16S rRNA gene was amplified with primers 338F (5′-ACTCCTACGGGAGGCAGCAG-3′) and 806R (5′-GGACTACHVGGGTWTCTAAT-3′) by thermocycler PCR system (GeneAmp 9700, ABI, United States). PCR products were purified and quantified, and next-generation sequencing was carried out using an Illumina MiSeq 2500 platform (Illumina, Inc., San Diego, CA, United States), and 250-bp paired-end reads were generated.

#### Processing and Analysis of Sequencing Data

The filtered reads were assembled into tags according to overlaps between paired-end reads with more than 10-bp overlap and less than 2% mismatch. Redundant tags were removed by software MOTHUR (Schloss et al., 2009) to obtain unique tags. The resultant unique tags were then employed to determine the abundance. The high-quality sequences were clustered into operational taxonomic units (OTUs) defined at a similarity of 97%. Diversity metrics were determined using the core-diversity plugin within QIIME2^1^ (Callahan et al., 2016). Feature level alpha diversity indices, including observed OTUs, Chao1 richness estimator, Shannon diversity index, and Faith’s phylogenetic diversity (PD) index, were estimated to assess the microbial diversity within an individual sample. Beta diversity was analyzed to assess the structural variation of microbiota across specimens, and then non-metric multidimensional scaling (NMDS) was determined (Vázquez-Baeza et al., 2013). Appropriate methods LEfSe were employed to identify the bacteria with different abundances among samples and groups (Segata et al., 2011). Unless specified above, parameters used in the analysis were set as default. The sequencing data were deposited in the Sequence Read Archive (SRA) under the accession number PRJNA636989.

### Statistical Analysis

The additives of silage were subjected to a completely randomized design, which was analyzed using the general linear model (GLM) of SAS (Statistical Analysis Software (SAS), 1996). Differences among various treatments were assessed using the probability of difference. Significant differences were compared using Duncan’s multiple range tests, and *P* < 0.05 was regarded as statistically significant.

## Results and Discussion

### Chemical and Microbial Compositions of CF

Table 1 presents the chemical and microbial compositions of CF. In this work, the levels of NDF and ADF were higher compared with previous reports, while the contents of DM, OM, CP, and EE were lower or comparable to previous reports (Régnier et al., 2013; Li et al., 2019d). Based on these data, CF had high protein content and moderate fiber content, and therefore, could be considered a high-quality roughage. WSC content plays a key role in evaluating fermentation quality. A WSC content (5.24%) lower than 6–7% DM is the theoretical threshold for well-preserved silage (Smith, 1962), and higher contents higher may lead to poor quality of CF silage. Our previous study confirmed that CF ensiled alone cannot achieve high fermentation quality (Li et al., 2019d). Besides, well-preserved silage needs a LAB number of more than 10^5^CFU/g of FM (Cai et al., 1998). The number of LAB, coliform, yeast, and mold in the fresh CF was 3.89, 1.53, 2.06, and 0 Log_10_ CFU/g (FM), respectively. Thus, LAB and yeast would grow when fresh CF was sealed in the bag, but the LAB counts were relatively low, while the counts for undesirable microorganisms were relatively high, and it cannot ensure desirable silage quality. This indicated that silage additives, such as LAB inoculants, CA, and MA, were necessary for CF silage preparation.

**TABLE 1:** Chemical and microbial composition of cassava foliage.

	Cassava foliage
Dry matter (% DM)	14.56
Organic matter (% DM)	90.33
Ether extract (% DM)	5.24
Water-soluble carbohydrate (% DM)	5.21
Crude protein (% DM)	18.11
Neutral detergent fiber (% DM)	42.02
Acid detergent fiber (% DM)	31.84
Lactic acid bacteria (Log_10_ CFU/gFM)	3.89
Coliform (Log_10_ CFU/gFM)	1.53
Yeast (Log_10_ CFU/gFM)	2.06
Mold (Log_10_ CFU/gFM)	0

*DM, dry matter; FM, fresh matter; CFU, colony forming unit.*

### CF Fermentation Quality

Tables 2, 3 present the fermentation characteristics of experimental silage. Additives enhanced the lactic acid content, reduced the pH value, and the contents of propionic acid, butyric acid, and ammonia-N, compared with the CK group. Silage pH is the most critical index for assessing fermentation quality, and a pH of 4.2 or lower is considered well-fermented silage (Edwards and McDonald, 1978).

**TABLE 2:** Fermentation quality of ensiled CF in the presence of CA and LAB.

Treatments	pH	(% DM)				
		Lactic acid	Acetic acid	Propionic acid	Butyric acid	Ammonia-N
CK	4.69a	3.72d	1.78a	1.64a	0.15a	2.09a
LAB	4.36b	4.14c	1.83a	1.27b	0.00b	1.36b
CA	4.03c	9.02b	1.78a	1.13b	0.00b	1.03c
CAL	4.02c	13.31a	1.78a	0.72c	0.00b	0.76d
SEM	0.16	1.99	0.01	0.22	0.04	0.27
*P*-value	<0.001	<0.001	0.90	<0.001	<0.001	0.02

*CK, control; LAB, *Lactobacillus plantarum*; CA, citric acid; CAL, citric acid + *Lactobacillus plantarum*, respectively; SEM, standard error of means. Means within the same column with different letters are significantly different (*P* < 0.05).*

**TABLE 3:** Fermentation quality of ensiled CF in the presence of MA and LAB.

Treatments	pH	(% DM)				
		Lactic acid	Acetic acid	Propionic acid	Butyric acid	Ammonia-N
CK	4.69a	3.72d	1.78a	1.64a	0.15a	2.09a
LAB	4.36b	4.14bc	1.83a	1.27b	0.00b	1.36b
MA	4.06c	4.39b	1.78a	1.04b	0.00b	1.17bc
MAL	4.04c	11.97a	1.78a	0.60c	0.00b	0.87d
SEM	0.15	1.98	0.01	0.22	0.04	0.26
*P*-value	<0.001	<0.001	0.81	<0.001	<0.001	0.014

*CK, control; LAB, *Lactobacillus plantarum*; MA, malic acid; MAL, malic acid + *Lactobacillus plantarum*; SEM, standard error of means. Means within the same column with different letters are significantly different (*P* < 0.05).*

The pH in all treatment groups was dramatically reduced after fermentation (*P* < 0.05), and the pH values of the additive-treated groups were below 4.2 (except for the LAB group), ensuring the good preservation of CF silage. Li et al. (2016) and He et al. (2019) have shown a similar pH of CA-treated silage, while the higher pH of MA-treated silage has been reported by Ke W. et al. (2018) and Ke W. C. et al., 2018. The lactic acid content in all treatments groups was remarkably elevated (*P* < 0.05). Moreover, the lactic acid content of the CAL and MAL groups was higher compared with the other groups (*P* < 0.05). These findings were consistent with previous data, indicating that CAL can increase the lactic acid content, while MAL has various effects (Ke W. C. et al., 2018). The acetic acid content of all groups was similar. Besides, the propionic acid content of the additive-treated groups was lower compared to the CK group, and the CAL and MAL groups had the lowest propionic acid content (*P* < 0.05). Furthermore, butyric acid was not detected in any of the additive-treated groups, indicating that additive-treated CF is well preserved, which is consistent with Lv et al. (2020). The ammonia-N content in the additive-treated groups was remarkably reduced, and the lowest ammonia-N content was observed in the CAL and MAL groups. Ke W. C. et al. (2018) have shown a similarly reduced ammonia-N in alfalfa silage in the presence of CAL or MAL. The comparatively low ammonia-N content in the additive-treated silage may be due to lower pH values, which can inhibit protease activity, resulting in better nutrient preservation. These results revealed that the addition of CA, MA, and *Lactobacillus plantarum* in the ensiling process could promote the fermentation quality, and the combination treatment could enhance the fermentation quality more efficiently.

### Microbial Community of CF Silage

A total of 901,998 raw reads and 680,140 filtered numeric reads were generated, and on average, 37,761 merged numeric read and 36,393 clean reads were obtained from each silage sample.

**FIGURE 1:**
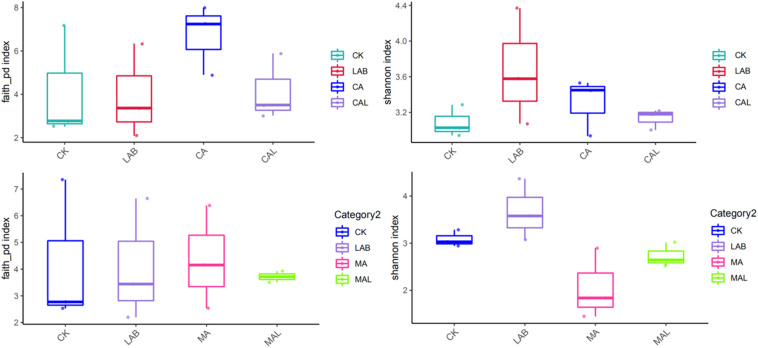
Alpha bacterial diversity of CF silage. CK, control; LAB, *Lactobacillus plantarum*; MA, malic acid; MAL, malic acid + *Lactobacillus plantarum*; CA, citric acid; CAL, citric acid + *Lactobacillus plantarum*.

**FIGURE 2:**
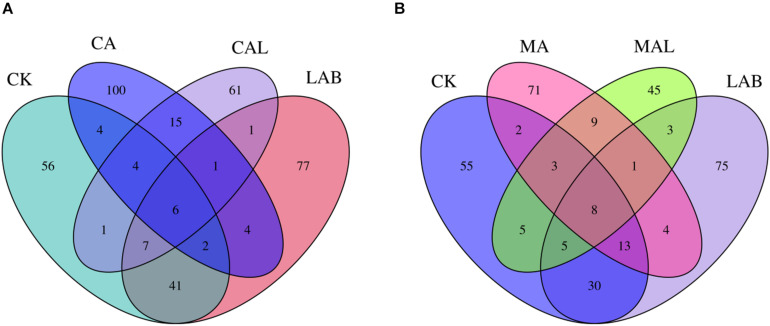
Venn analysis of OTUs for CF silage treated with CA and *Lactobacillus plantarum* **(A)** or MA and *Lactobacillus plantarum* **(B)**. CK, control; LAB, *Lactobacillus plantarum*; MA, malic acid; MAL, malic acid + *Lactobacillus plantarum*; CA, citric acid; CAL, citric acid + *Lactobacillus plantarum*.

**FIGURE 3:**
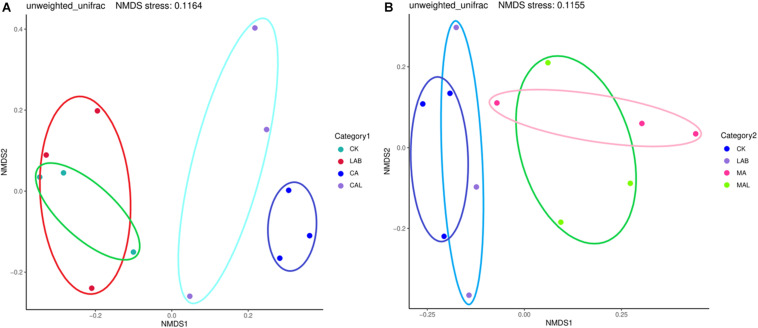
Representation of microbial beta diversity of CF silage treated with CA and *Lactobacillus plantarum* **(A)**, or MA and *Lactobacillus plantarum* **(B)**. CK, control; LAB, *Lactobacillus plantarum*; MA, malic acid; MAL, malic acid + *Lactobacillus plantarum*; CA, citric acid; CAL, citric acid + *Lactobacillus plantarum*.

Figure 1 shows the alpha diversity of the microbial community in each silage sample. The Faith’s PD and Shannon indices of bacterial diversity were affected by additive treatment. For community richness comparison, the indices of Faith’s PD and Shannon were relatively higher in the LAB and CA groups but lower in the MAL and CAL groups, suggesting different microbial diversity. Nevertheless, no significant difference in alpha diversity was observed among all treatments. Figure 2 shows a Venn analysis of OTUs for CF silage treated with CA and *Lactobacillus plantarum* (A) or MA and *Lactobacillus plantarum* (B). The four treatments contained six common OTUs (Figure 2A), and there were 56,77,100, and 61 unique OTUs in the CK, LAB, CA, and CAL groups, respectively. Meanwhile, the CK, LAB, MA, and MAL treatments contained eight common OTUs (Figure 2A), and 55, 75, 71, and 45 unique OTUs, respectively. The NMDS was employed to examine the correlations among the community structures of the silage microbial community. The results showed that there was a clear separation and difference of bacterial communities in the ensiled groups (Figures 3A,B), suggesting that the microbiota was altered during the ensiling process in the presence of different additives. Such a difference in silage quality may be attributed to the variation of the microbial community (Ni et al., 2017; Dong et al., 2019; Wang et al., 2019). Therefore, based on alpha and beta diversity analyses, we concluded that the CA, MA, and *Lactobacillus plantarum* treatments could impair the microbial diversity and community structure of CF silage.

**FIGURE 4:**
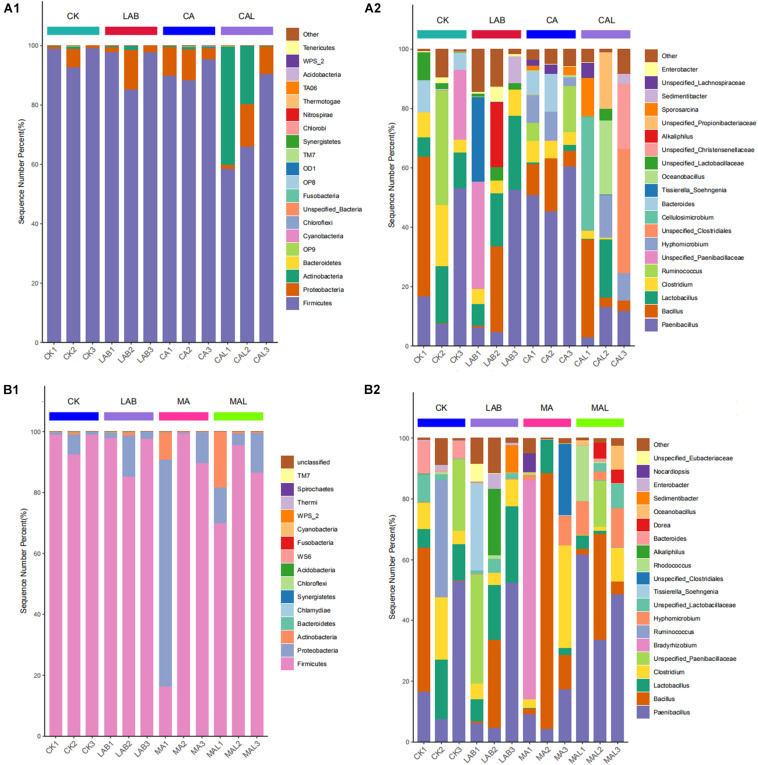
Relative abundances of the bacterial phyla **(A1,B1)** and genera **(A2,B2)** in CF silage treated with CA and *Lactobacillus plantarum*, or MA and *Lactobacillus plantarum*. CK, control; LAB, *Lactobacillus plantarum*; CA, citric acid; CAL, citric acid + *Lactobacillus plantarum*, MA, malic acid; MAL, malic acid + *Lactobacillus plantarum*.

Figures 4A1,B1 describes the microbiota of all samples according to the distribution of DNA sequences at the phylum level. Firmicutes and Proteobacteria predominated in all the groups, accounting for more than 98% of the total sequences, and the abundance shifted following the ensiling treatments. The abundance of Firmicutes was lower, while the abundances of Proteobacteria and Actinobacteria were higher in the additive-treated groups compared with the CK group. Xu et al. (2017) and Dong et al. (2019) have reported similar results in corn stover and red clover silage. Besides, the abundance of Actinobacteria was significantly higher in the CAL and MAL groups.

To further understand the effects of CA, MA, and *Lactobacillus plantarum* on the microbial community during ensiling, we examined microbiota structures of CF silages at the genus level (Figures 4A2,B2). *Paenibacillus* and *Bacillus* were the predominant strains in all groups. *Lactobacillus*, *Clostridium*, *Ruminococcus*, unspecified_*Paenibacillaceae*, and *Bacteroides* were the sub-dominant microbes in the CK group. *Lactobacillus*, *Clostridium*, unspecified_*Paenibacillaceae*, *Tissierella_Soehngenia*, and *Alkaliphilus* were the sub-dominant microbes in the LAB group. *Clostridium*, *Ruminococcus*, *Hyphomicrobium*, and *Bacteroides* were the sub-dominant microbes in the CA group. *Clostridium*, *Hyphomicrobium*, unspecified_*Clostridiales*, *Cellulosimicrobium*, *Oceanobacillus*, unspecified_*Christensenellaceae*, unspecified_*Propionibacteriaceae*, and *Sporosarcina* were the sub-dominant microbes in the CAL group, *Lactobacillus*, *Clostridium*, *Bradyrhizobium*, *Hyphomicrobium*, and unspecified_*Clostridiales* were the sub-dominant microbes in the MA group, *Lactobacillus*, *Clostridium*, Unspecified_*Paenibacillaceae*, *Hyphomicrobium*, unspecified_*Lactobacillaceae*, *Rhodococcus*, *Dorea*, and *Oceanobacillus* were the dominant microbes in the MAL group.

*Paenibacillus* was one of the dominant microbes in all groups, which was rarely reported in silage microorganisms. Ash et al. (1993) have proposed to separate 11 species from *Bacillus*, which is usually Gram-negative, and establish a new genus *Paenibacillus*, which is Gram-positive. They have also found that *Paenibacillus* is facultatively anaerobic and can produce organic acids, such as lactic acid, by using various sugars. Therefore, it is a desirable bacterial strain in silage. Bacillus is also a type of Gram-positive bacteria, that can produce bacteriocin and inhibit pathogenic bacteria (Bizani and Brandelli, 2002). Besides, *Bacillus* is usually facultatively anaerobic, and some species of *Bacillus* also produce lactic acid (Liu et al., 2008). Therefore, by inhibiting undesirable bacteria and producing lactic acid in silage, *Bacillus* has a positive effect on CF silage fermentation. *Lactobacillus* was dominant in the CK and LAB groups, while its abundance was lower in the CA, MA, CAL, and MAL groups. This may be because CA and MA reduce pH, which may affect the activity of *Lactobacillus.* Moreover, *Paenibacillus* and *Bacillus* competed with it for fermentation substrate, further hindering the growth of *Lactobacillus.* In contrast, Lv et al. (2020) have reported that the abundance of *Lactobacillus* is increased with the increase of CA addition ratio, and this difference may be because of the different silage material and the amount used. *Clostridium* is an undesirable bacterial strain because it consumes sugars and proteins to produce butyric acid, which reduces the fermentation quality (Li et al., 2019d). In this study, the abundance of *Clostridium* was relatively higher in the CK and LAB groups, and CA, CAL, and MAL treatments decreased the abundance of *Clostridium*, which is consistent with the higher lactic acid content and absence of butyric acid in these silages. Similarly, Li et al. (2019d) have reported that formic acid can hamper the *Clostridium* in bur clover and annual ryegrass silage.

Furthermore, the abundances of *Cellulosimicrobium*, *Hyphomicrobium*, and *Oceanobacillus* were obviously increased in the CAL and MAL groups compared with the CK and LAB groups. *Cellulosimicrobium* is a Gram-positive actinobacterial strain that can secrete enzymes using different carbon sources and then produce organic acids (Li et al., 2008; Hamada et al., 2016; Dou et al., 2019). Martineau et al. (2013) have reported that *Hyphomicrobium* can use nitrate as a nitrogen source. Nitrate is a known product of undesirably fermented silage, and the higher abundance of *Hyphomicrobium* in CA, MA, CAL, and MAL silages may improve the fermentation quality. *Oceanobacillus* is also a Gram-positive bacterial strain that was isolated from the ocean (Lu et al., 2001). Besides, *Oceanobacillus* is close to the genus *Bacillus* and found in fermented foods. Therefore, we speculated that these above-mentioned strains had a beneficial effect on silage fermentation. In general, the three microorganisms are rarely reported in silage. In our current work, the presence of the above-mentioned strains in the silage resulted in improved fermentation quality, and the function of these microbes need to be further studied.

**FIGURE 5:**
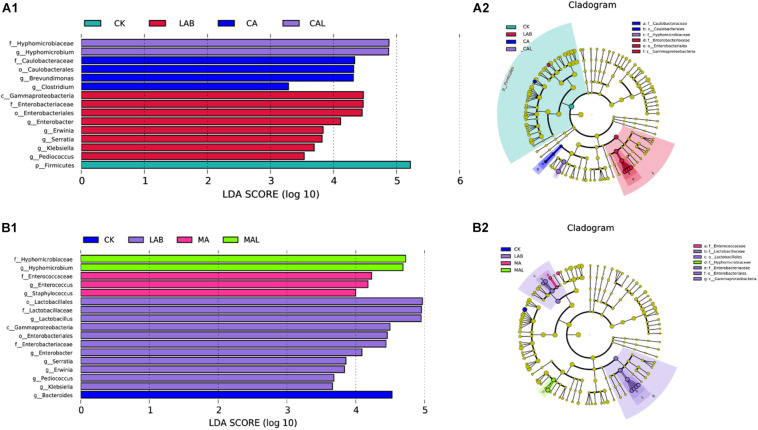
Comparison of microbial variations using the LEfSe online tool for CF silage treated with CA and *Lactobacillus plantarum* **(A1,A2)**, or MA and *Lactobacillus plantarum* **(B1,B2)**. CK, control; LAB, *Lactobacillus plantarum*; MA, malic acid; MAL, malic acid + *Lactobacillus plantarum*; CA, citric acid; CAL, citric acid + *Lactobacillus plantarum*.

The linear discriminant analysis (LDA) effect size (LEfSe) method was adopted to examine the differences in microbial communities between groups and explore the specific bacteria in each group (LDA score > 3.0). Figures 5A1,A2 shows that CA and LAB exerted a dramatic impact on the microbial community. Firmicutes were the most abundant phylum in the CK group. *Gammaproteobacteria* was the most abundant class, *Enterobacteriaceae* was the most abundant family, *Enterobacteriales* was the most abundant order, and *Enterobacter*, *Erwinia*, *Serratia*, *Klebsiella*, and *Pediococcus* were the most abundant genera in the LAB group. *Caulobacteriaceae* was the most abundant family, *Caulobacterales* was the most abundant order, and *Brevundimonas* and *Clostridium* were the top two abundant genera in the CA group. *Hyphomicrobiaceae* was the most abundant family, and *Hyphomicrobium* was the most abundant genus in the CAL group. The impacts of MA and LAB on the microbial community are depicted in Figures 5B1,B2. *Bacteroides* was the most abundant genus in the CK group. *Gammaproteobacteria* was the most abundant class, *Lactobacillaceae* and *Enterobacteriaceae* were the most abundant families, *Lactobacillales* and *Enterobacteriales* were the most abundant orders, and *Lactobacillus*, *Enterobacter*, *Erwinia*, *Serratia*, *Klebsiella*, and *Pediococcus* were the most abundant genera in the LAB group. *Enterobacteriaceae* was the most abundant family, and *Enterococcus* and *Staphylococcus* were the most abundant genera in the MA group. *Hyphomicrobiaceae* was the most abundant family, and *Hyphomicrobium* was the most abundant genus in the MAL group. These microorganisms could be used as biomarkers of the silages produced by the different treatments. Different treatments greatly affected silage microbial diversity. The community of CA was similar to MA, and the community of CAL was also similar to MAL, in turn affecting the fermentation quality of the silage, which was better than that in the CK and LAB group. Wang et al. (2019) have used the LEfSe method to assess the differences in the microbiome of silage and shown a significant correlation with silage fermentation.

## Conclusion

The addition of CA, MA, and LAB significantly altered the bacterial community of CF silage and improved the fermentation quality. The additives reduced the pH, butyric acid, and ammonia-N while increasing the lactic acid content. The CAL and MAL combination treatments showed similarly remarkable effects. The organic acid-producing bacteria *Paenibacillus* and *Bacillus* were the predominant strains in CF silage, the addition of CA and MAL increased the abundance of *Paenibacillus*, and the addition of MA increased the abundance of *Bacillus*. Moreover, the combination treatments of CAL and MAL increased the abundances of *Cellulosimicrobium*, *Hyphomicrobium*, and *Oceanobacillus*, which are potentially desirable bacteria. The above-mentioned findings proved that CA and MA could enhance the silage quality of CF, and their combinations with LAB were more effective.

## Data Availability Statement

The datasets presented in this study can be found in online repositories. The names of the repository/repositories and accession number(s) can be found below: http://www.ncbi.nlm.nih.gov/sra/, PRJNA636989.

## Author Contributions

ML, LZ, XZ, and HZ did the experimental design work. ML, LZ, RL, and JT conducted the experiments. ML, LZ, QZ, XZ, HZ, RL, and JT collected and analyzed the data. ML and XZ wrote the manuscript. All authors read and approved the manuscript.

## Conflict of Interest

The authors declare that the research was conducted in the absence of any commercial or financial relationships that could be construed as a potential conflict of interest.

## Footnotes

1 https://docs.qiime2.org/2019.1/
